# Outcomes of kidney transplantation from elderly deceased donors of a Korean registry

**DOI:** 10.1371/journal.pone.0232177

**Published:** 2020-06-11

**Authors:** Heungman Jun, Yeong Hoon Kim, Joong Kyung Kim, Chan-Duck Kim, Jaeseok Yang, Curie Ahn, Sang Youb Han, the Korean Organ Transplantation Registry Study Group

**Affiliations:** 1 Department of Surgery, Inje University College of Medicine, Ilsan Paik Hospital, Goyang, Korea; 2 Department of Internal Medicine, Inje University College of Medicine, Busan Paik Hospital, Busan, Korea; 3 Division of Nephrology, Department of Internal Medicine, Bong Seng Memorial Hospital, Busan, Korea; 4 Department of Internal Medicine, School of Medicine, Kyungpook National University, Kyungpook National University Hospital, Daegu Korea; 5 Department of Surgery, Seoul National University Hospital, Seoul, Korea; 6 Transplantation Research Institute, Seoul National University College of Medicine, Seoul, Korea; 7 Transplantation Center, Seoul National University Hospital, Seoul, Korea; 8 Department of Internal Medicine, Seoul National University Hospital, Seoul National University College of Medicine, Seoul, Korea; 9 Department of Internal Medicine, Inje University College of Medicine, Ilsan Paik Hospital, Goyang, Korea; Imperial College Healthcare NHS Trust, UNITED KINGDOM

## Abstract

To overcome organ shortage, expanded criteria donors, including elderly deceased donors (DDs), should be considered. We analyzed outcomes of kidney transplantation (KT) from elderly DDs in a nationwide study. In total, data of 1049 KTs from DDs using the database of Korean Organ Transplantation Registry (KOTRY) were retrospectively analyzed based on the age of DDs: age ≥60 years vs. <60 years. Clinical information, graft status, and adverse events were reviewed in DDs and recipients. The mean age of the 1006 DDs was 51.04±10.54 years, and 21.5% of donors were aged ≥60 years. Elderly DDs had a significantly higher prevalence of diabetes and hypertension and higher Kidney Donor Risk Index (KDRI) and Kidney Donor Profile Index (KDPI). The mean age of the recipients was 47.45±14.87 years. Patients who received KT from elderly DDs were significantly older (53.12±15.14 vs. 45.88±14.41, P<0.001) and had a higher rate of diabetes (41.9 vs. 24.4%, P<0.001). Graft outcomes were not significantly different. Renal function was similar between the groups at the time of discharge and at 6 months, 1 year, and 2 years after KT. The rate of delayed graft function (DGF) was not significantly different. Risk factors of DGF were significantly different in DDs aged ≥60 years and <60 years. In the multivariable model, male sex (odds ratio: 3.99, 95% confidence interval: 1.42–11.22; P = 0.009) and KDRI (12.17, 2.23–66.34; P = 0.004) were significant risk factors for DGF in DDs aged ≥60 years. In DDs aged <60 years, thymoglobulin induction (2.62, 1.53–4.48; P<0.001) and continuous renal replacement therapy (3.47, 1.52–7.96; P = 0.003) were significant factors. Our data indicated that graft outcomes, including renal function and DGF, were similar for elderly DDs and DDs aged <60 years. Elderly DDs might be considered tolerable donors for KT, with active preoperative surveillance.

## Introduction

Many dialysis patients die while waiting for kidney transplantation (KT) due to a shortage of kidneys. In Korea, 5.2 patients waiting for KT die each day [1]. To overcome organ shortage, expanded criteria donors (ECDs) [2], including elderly deceased donors (DDs), should be considered. The rate of KTs from elderly DDs has increased in many countries [3].

Although patient prognosis is controversial, many researchers have reported that KT from elderly DDs is associated with delayed graft function (DGF) and lower graft survival [2, 4]. Thus, KT from elderly DDs has been performed on a limited number of elderly patients or patients with a lower life expectancy.

The Kidney Donor Risk Index (KDRI) and Kidney Donor Profile Index (KDPI) scoring systems for DDs are widely used to predict postoperative graft function [5]. In these scoring systems, the discard rate is increased because old age is one of the strongest negative risk factor for graft function [6]. As shown in previous reports, a small number of elderly DDs have been included, and a few reports have shown that the renal outcomes from elderly DDs are not worse than those from any other DD in a different age group. Therefore, this study aimed to clarify the outcomes of KT from elderly DDs in a nation-wide large population.

## Materials and methods

### KOTRY design and ethical considerations

This study was based on data from the Korean Organ Transplant Registry (KOTRY). The KOTRY was established in 2014 and has been managing nationwide cohorts of kidney, liver, pancreas, heart, and lung transplant patients [7]. These cohorts include demographic and clinical data of both donors and recipients. After approval, the data are available as per the proposal of the investigator. The data do not include any personal information. This study was approved by the Institutional Review Board of Ilsan-Paik Hospital (No. 2018-03-020).

### Data collection

One thousand forty-nine DD KTs conducted between April 2014 and December 2016 from the KOTRY database of 30 renal transplant centers were reviewed retrospectively. Forty-three of the 1049 DDs were excluded owing to donation after cardiac death (DCD), and the remaining 1006 were included in this study. Data from DDs included patient demographics, occurrence of comorbidities, cause of brain death, and laboratory tests, which were used especially for KDPI calculations. Data from the recipients included demographics, occurrence of comorbidities, laboratory tests, immunosuppressive regimens, graft status (including allograft rejection), DGF, graft loss, and adverse events, including urine leakage, bleeding, arterial thrombosis, and lymphocele.

### Statistical analyses

All donors were stratified into two groups based on age: more than 60 years and below. In this study, donors aged over 60 years were defined as elderly. Univariable analysis was performed using the Mann-Whitney U test and Spearman correlation, based on the characteristics of the donors and recipients in the two groups. Logistic regression analysis was conducted to analyze the odds ratio (OR) of DGF between ages ≥60 years and <60 years. Crude and adjusted analyses were performed, and 95% confidence intervals (CIs) were calculated. All statistical analyses were performed using the Statistical Package for Social Science (SPSS) software version 25 (IBM Corp, United States). A P*-*value of <0.05 was considered statistically significant.

## Results

### Baseline characteristics of the DDs

**Fig 1:**
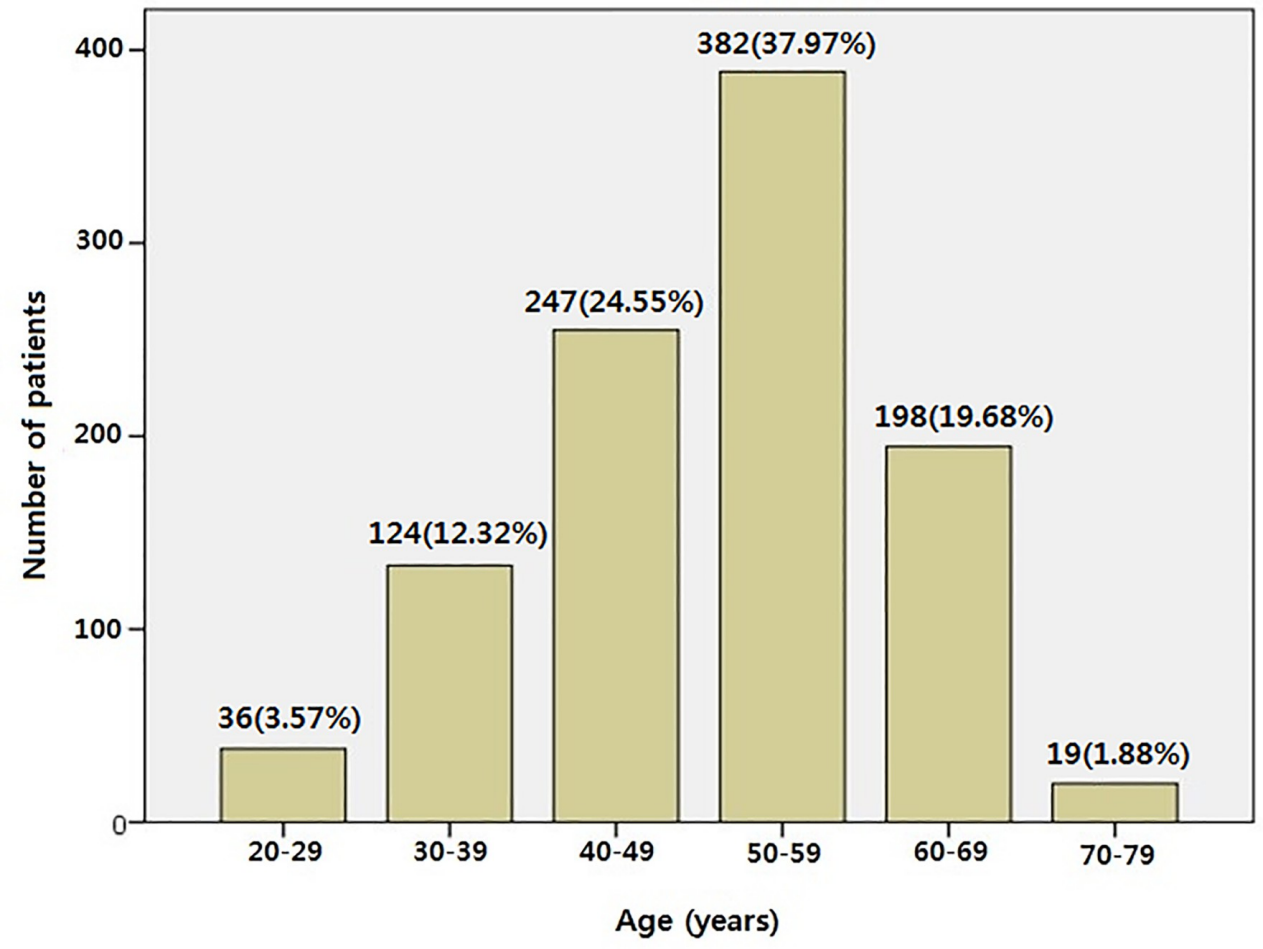
Age distribution of deceased donors.

The mean age of the 1006 DDs was 51.04±10.54 years; 198 (19.67%) were ≥60 but <70 years old, and 19 (1.88%) were ≥70 years old (Fig 1). Furthermore, 121 (12.0%) DDs were diagnosed with diabetes, 248 (24.7%) with hypertension, 55 (5.5%) with continuous renal replacement therapy (CRRT), and 22 (2.2%) with extracorporeal membrane oxygenation (ECMO). Causes of brain death were cerebrovascular accident, head trauma, and anoxia (40.9%, 33.1%, and 20.9%, respectively). The serum creatinine (sCr) level just before surgery was 1.67 ± 3.39 mg/dL, and the mean KDRI and KDPI were 1.24 ± 0.40 and 63.58 ± 25.16, respectively.

The age distribution of the DDs was as follows: 36 (3.57%): 20–39 years, 124 (12.32%): 30–39 years, 247 (24.55%): 40–49 years, 382 (37.97%): 50–59 years, 198 (19.67%): 60–69 years, and 19 (1.88%): ≥70 years.

A total of 217 (21.57%) DDs were aged ≥60 years. Elderly DDs had significantly different characteristics compared to DDs aged <60 years; specifically, they had a higher prevalence of diabetes and hypertension, as well as higher KDRI and KDPI (Table 1).

**Table 1:** Baseline characteristics of deceased donors, based on age, for kidney transplantation.

Characteristics	D ≥60 years (n = 217)	D <60 years (n = 789)	P value
Male	133 (61.2)	469 (59.4)	0.640
Age, years	64.01 ± 3.49	47.47 ± 8.90	<0.001
Use CRRT	14 (6.4)	41 (5.1)	0.500
Use ECMO	5 (2.3)	17 (2.1)	0.799
Diabetes	38 (17.5)	83 (10.5)	0.018
Hypertension	73 (33.6)	175 (22.1)	0.001
Cause of brain death			
Head trauma	79 (36.4)	254 (32.1)	
CVA/stroke	97 (44.7)	314 (39.7)	
Anoxia	25 (11.5)	185 (23.4)	
CNS tumor	0 (0)	4 (0.5)	
Other	16 (7.3)	32 (4.0)	
Cold ischemic time, min	284.53 ± 144.09	301.58 ± 427.70	0.630
Body mass index, kg/m^2^	26.28 ± 15.69	25.31 ± 12.87	0.349
Preoperative sCr (mg/dL)	1.54 ± 1.22	1.70 ± 3.70	0.542
KDRI	1.43 ± 0.50	1.19 ± 0.35	<0.001
KDPI	72.70 ± 24.64	61.07 ± 24.74	<0.001

Data are expressed as numbers (%) and means ± SDs.

CRRT, continuous renal replacement therapy; ECMO, extracorporeal membrane oxygenation; CVA, cerebrovascular accident; CNS, central nervous system; KDRI, Kidney Donor Risk Index; KDPI, Kidney Donor Profile Index; sCr, serum creatinine.

### Baseline characteristics of the recipients

The mean age of the recipients was 47.45 ± 14.87 years; 284 (28.2%) had diabetes, 93 (9.2%) had multiple KT, and 8 (0.8%) had a dual transplant. The mean human leukocyte antigen (HLA) mismatch was 3.50 ± 1.82, and thymoglobulin induction therapy was performed in 294 (29.2%) recipients. The patients who received KT from elderly DDs were significantly older (53.12±15.14 vs. 45.88±14.41, P<0.001) and had a higher rate of diabetes (41.9 vs. 24.4%, P<0.001) (Table 2).

**Table 2:** Baseline characteristics of recipients from deceased donors, based on age, for kidney transplantation.

Characteristics	D ≥60 years (n = 217)	D <60 years (n = 789)	P value
Male	146 (67.2)	575 (72.8)	0.107
Age, years	53.12 ± 15.14	45.88 ± 14.41	<0.001
Waiting time*, months	84.12 ± 106.09	91.33 ± 73.05	0.248
Diabetes	91 (41.9)	193 (24.4)	<0.001
Multiple kidney transplant	10 (4.6)	83 (10.5)	0.008
Dual transplant	2 (0.9)	6 (0.7)	0.685
Body mass index, kg/m^2^	23.73 ± 5.79	23.08 ± 5.79	0.145
HLA mismatch	3.70 ± 1.80	3.45 ± 1.82	0.076
Thymoglobulin induction	68 (31.3)	226 (28.6)	0.652

Data are expressed as numbers (%) and means ± SDs.

HLA, human leukocyte antigen; SD, standard deviation.

*Waiting time means the time between the first dialysis and transplantation.

### Graft outcomes

There were no significant differences in graft outcomes between the groups. Renal function was similar between two groups at the time of discharge and 6 months, 1 year, and 2 years after KT (Table 3). The mean duration of follow-up in the recipients was 25.51 ± 11.78 months. At the time of discharge, the sCr levels were 1.36 ± 0.85 mg/dL and 1.39 ± 0.93 mg/dL in DDs aged ≥60 years and DDs aged <60 years, respectively. The sCr levels were 1.23 ± 0.43 mg/dL and 1.25 ± 0.59 mg/dL in DDs aged ≥60 years and DDs aged <60 years, respectively, two years after KT.

**Table 3:** Graft outcomes of recipients from deceased donors, based on age, for kidney transplantation.

Characteristics	D ≥60 years (n = 217)	D <60 years (n = 789)	P value
sCr level at discharge, mg/dL	1.36 ± 0.85	1.39 ± 0.93	0.689
Delayed Graft Function	23 (10.5)	63 (8.0)	0.220
sCr at POD 6M, mg/dL	1.32 ± 0.52	1.27 ± 0.54	0.190
sCr at POD 1Y, mg/dL	1.25 ± 0.43	1.23 ± 0.52	0.733
sCr at POD 2Y	1.23 ± 0.43	1.25 ± 0.59	0.787
Graft loss	17 (7.8)	40 (5.0)	0.135

Data are expressed as numbers (%) and means ± SDs.

POD, postoperative day; M, months; Y, years; SD, standard deviation.

The graft survival rate was not significantly different between the two groups. The one-year and death-censored graft survival rates in DDs ≥60 years were 93.5% and 95.8%, respectively; the corresponding rates in DDs <60 years were 96.1% and 97.2%, respectively. The two-year and death-censored graft survival rates in DDs ≥60 years were 92.1% and 94.9%, respectively, and 94.9% and 96.3%, respectively, in DDs <60 years.

In particular, there were no significant differences in the DGF rate between DDs ≥60 years and <60 years; these rates were 23/217 (10.5%) and 63/789 (8.0%), respectively, P = 0.220. However, graft loss tended to be higher in the elderly group; 7.8% vs. 5.0%, P = 0.135 (Table 3). The OR of DGF between ages ≥60 years and <60 years was 1.38 (95% CI, 0.84–2.26; P = 0.224). There were no significant differences in surgical complications including urine leakage, bleeding, vascular thrombosis, wound problems, and lymphocele.

According to the univariable analysis, risk factors of DGF were significantly different in DDs ≥60 years and <60 years. Males had a higher DGF rate in the elderly population, but there was no difference in DDs <60 years. The KDRI was significantly higher in DDs ≥60 years, as expected. However, the KDPI was not different between the DGF group and non-DGF group. The sCr level was significantly different, but only in DDs <60 years. As expected, the rate of DGF was higher in patients who used CRRT, or those with a higher preoperative sCr level. CRRT was administered to 55 (5.4%) DDs. The prevalence of DGF was significantly higher in DDs with CRRT than in those without (21.8% vs. 7.7%, P = 0.001); however, the postoperative sCr levels and graft loss were not significantly different. Furthermore, it was higher in patients who had thymoglobulin induction in DDs <60 years (Table 4).

**Table 4:** Univariable analysis of delayed graft function in deceased donors, based on age.

	D ≥60 years (n = 217)		D <60 years (n = 789)	
Characteristics	DGF (n = 23)	No DGF (n = 194)	DGF (n = 63)	No DGF (n = 726)
Male (D)	9 (39.1)*	124 (63.9)	37 (58.7)	432 (59.5)
Use CRRT (D)	1 (4.3)	11 (5.6)	9 (14.2)*	32 (4.4)
Use ECMO (D)	0 (0)	5 (2.5)	2 (3.1)	15 (2.0)
Diabetes (D)	5 (21.7)	33 (17.0)	4 (6.3)	79 (10.8)
Hypertension (D)	11 (47.8)	62 (31.9)	12 (19.0)	163 (22.4)
CIT, min	267.6 ± 98.61	286.1 ± 147.8	325.1 ± 129.9	299.7 ± 442.6
Preoperative sCr (D)	1.86 ± 1.09	1.51 ± 1.23	2.69 ± 1.80*	1.62 ± 3.89
KDRI (D)	1.82 ± 0.77*	1.39 ± 0.44	1.31 ± 0.32*	1.18 ± 0.35
KDPI (D)	80.78 ± 21.55	71.74 ± 24.86	67.80 ± 21.14*	60.57 ± 24.97
Male (R)	7 (30.4)	64 (32.9)	48 (76.1)	527 (72.5)
Diabetes (R)	7 (30.4)	84 (43.2)	17 (26.9)	176 (24.2)
Multiple transplant (R)	1 (4.3)	9 (4.6)	7 (11.1)	76 (10.4)
Dual transplant (R)	0 (0)	2 (1.0)	0 (0)	6 (0.8)
HLA mismatch	3.83 ± 1.85	3.65 ± 1.85	3.49 ± 1.82	3.45 ± 1.83
Thymoglobulin induction	8 (34.7)	60 (30.9)	32 (50.7)*	194 (26.7)

Data are expressed as numbers (%) and means ± SDs.

*P<0.05; (D) means in donors and (R) means in recipients.

DCD, donor after cardiac death; CRRT, continuous renal replacement therapy; ECMO, extracorporeal membrane oxygenation; CIT, cold ischemic time; sCr, serum creatinine; KDRI, Kidney Donor Risk Index; KDPI, Kidney Donor Profile Index; HLA, human leukocyte antigen; SD, standard deviation; DGF, delayed graft function.

In the multivariable model, male sex (OR 3.99, 95% CI 1.42–11.22; P = 0.009) and KDRI (12.17, 2.23–66.3; P = 0.004) were significant risk factors for DGF in DDs aged ≥60 years. In DDs aged <60 years, thymoglobulin induction (2.62, 1.53–4.48; P<0.001) and CRRT use (3.48, 1.52–7.96; P = 0.003) were significant factors (Table 5).

**Table 5:** Multivariable analysis with adjusted odds ratios for delayed graft function in deceased donors.

Characteristics	Adjusted OR	P value
D ≥60 years (n = 217)		
Male	3.99 (1.42–11.22)	0.009
KDRI	12.17 (2.23–66.3)	0.004
Serum creatinine (recipient)	1.42 (0.97–2.08)	0.070
Age (recipient)	1.0 (0.91–1.09)	1.000
KDPI	0.97 (0.92–1.02)	0.967
D<60 years (n = 789)		
Thymoglobulin induction	2.62 (1.53–4.48)	< 0.001
CRRT	3.48 (1.51–7.96)	0.003
KDRI	1.07 (0.32–3.64)	0.911
Serum creatinine (donor)	1.03 (0.99–1.07)	0.177
Male	0.94 (0.55–1.63)	0.836
Age (recipient)	1.02 (0.98–1.06)	0.253
KDPI	1.02 (0.97–1.06)	0.236

Data are expressed as odds ratio (95% confidence interval).

CRRT, continuous renal replacement therapy; KDRI, Kidney Donor Risk Index; KDPI, Kidney Donor Profile Index.

## Discussion

In our large, nation-wide study, graft outcomes, including renal function and DGF rate, were similar in elderly DDs compared to DDs aged <60 years. The two groups exhibited different risk factors for DGF. In elderly DDs, male sex and KDRI were risk factors, while KDPI was a significant risk factor for DGF in non-elderly DDs.

The graft outcomes of KT from elderly donors are still controversial. In living KT patients, graft outcomes in living donors aged ≥70 were worse than those in young living donors [8]. In DD KT, the outcomes also were not good. Marconi et al reported that outcomes of KT from DDs <70 years were better, compared to DDs >70 [9]. Lapointe et al studied KT in DDs >60 years and reported that the age of donors was related to the DGF rate [10]. However, several contradicting results have been reported. There was no significant difference in graft loss in KT from living donors older than 60 years [11]. Death-censored graft loss was not related to the age of the donor [12]. Several European studies also showed similar results between KT from donors >70 years of age and KT from extended criteria donors [13]. Our study findings aligned with these positive results. Although we analyzed the renal outcomes for two years, the early DGF rate was tolerable and renal function was also stable for these two years, compared to non-elderly DDs. Considering that the elderly DDs had higher rates of diabetes and hypertension, and higher KDPI and KDRI, this result could be more promising.

Various scoring systems for predicting graft outcomes after DD KT have been introduced for the allocation process. In 2009, the KDRI was developed for assessment and decision-making using donor factors, including age, prevalence of hypertension and diabetes, cause of death, and sCr level. The KDRI provided better information about the relative risk of kidney graft failure [5]. Following the KDRI, the KDPI currently plays an important role in the allocation process, in which decisions regarding DD kidneys are made [14,15]. The KDPI represents a percentile value of relative scores, compared to kidney grafts recovered in the prior calendar year [16]. In the Eurotransplant Senior Program, the KDRI reflects the graft outcomes of KT for donors >65 years of age [17]. In our study, the KDRI better represented DGF than KDPI in KT from DDs ≥60 years. A previous report indicated that the KDRI was better than the KDPI in estimating graft survival [18]. The KDPI is a relative mapping of the KDRI. [14] This principle difference between two indexes could lead to a different result in this study.

In addition to scoring systems, other efforts have been made to improve the decision-making process of KT from elderly DDs and marginal donors. Remuzzi et al reported that outcomes of KT from donors >60 years of age who underwent preimplantation biopsy-guided allocation were similar to those from young donors [4]. Based on this histological evaluation, dual transplantation may be considered in cases with insufficient nephron mass for sole transplantation [19]. It has recently been reported that stable transplant can be performed in DDs >80 years of age through biopsy-guided allocation [20].

Numerous efforts have been made to increase the graft pool and reduce waiting times. Longevity matching to provide kidneys of elderly DDs to elderly recipients is becoming commonplace. In the UK, particularly in the Cambridge group, the increased use of DCD and elderly DBD has reduced the waiting time in both, young and elderly recipients [21]. In the Korea registry, elderly recipients demonstrate shorter waiting times than young recipients; however, the difference is not statistically significant (Table 2). More aggressive donor selection including DCD expansion is needed.

Our study has a few limitations that should be addressed. Because groups were divided into two; younger and older than the age of 60, detailed comparisons between different age groups are not feasible at this point. In addition, long-term outcomes, including survival, could not be evaluated due to the short follow-up period. Regarding KDRI and KDPI, age plays an important role, and old age itself causes an increase in KDRI and KDPI [14]. Although efforts were made to evaluate the risk factors that were reflective of postoperative graft function in elderly DDs and establish an appropriate scaling system, it was not easy to clearly clarify these factors in this study. Although our results were based on a nation-wide registry data, each transplant institution has its own standard based on regionals circumstances. Future studies should include detailed comparisons between the age groups and a longer follow-up and evaluate long-term outcomes.

## Conclusions

Our study demonstrates that KT from elderly DDs ≥60 years show similar postoperative graft functions and DGF compared to that from non-elderly DDs. KTs from elderly DDs have few risk factors predictive of postoperative graft function, including KDRI. Therefore, elderly DDs might be considered tolerable donors, with active preoperative surveillance. In the future, we would like to present extensive research results, including recent information on elderly DDs.
